# Quality Evaluation Focusing on Tissue Fractal Dimension and Chemical Changes for Frozen Tilapia with Treatment by Tangerine Peel Extract

**DOI:** 10.1038/srep42202

**Published:** 2017-02-07

**Authors:** Qi He, Zhao Yang, Bin Gong, Jingjing Wang, Kaijun Xiao, Shang-Tian Yang

**Affiliations:** 1 College of Food Science and Engineering, South China University of Technology, Guangzhou city, Guangdong province, 510640, China; 2 Guangdong Food and Drug Vocational College, Guangzhou city, Guangdong province, 510520, China; 3 Department of Chemical and Biomolecular Engineering, The Ohio State University, Columbus, Ohio, 43210, US

## Abstract

This work aimed to establish an effective approach to evaluate the quality of frozen fish, focusing on changes in fish tissue structure and chemical composition during storage. Fresh tilapia samples were treated by coating with tangerine peel (TP) extract and then stored at −4, −8 and −18 °C, respectively, for 40 days. The frozen fish tissues were analyzed for structural and chemical changes. Fractal dimension, which quantifies the porous structure formed in the tissue samples, texture properties including hardness and springiness, and moisture content and water activity all decreased during the storage, while the extents of lipid oxidation, measured as peroxide value and thiobarbituric acid concentration, and protein degradation, monitored with total volatile basic nitrogen and trichloroacetic acid soluble peptides, increased. The change rates of these parameters decreased with decreasing the storage temperature and by applying TP extract. A model was developed for predicting fractal dimension, which indicated the quality of preserved tilapia and thus can be used to predict the shelf life under different storage temperatures. The results demonstrated that TP extract could extend the shelf life of frozen tilapia by 35–45% by inhibiting changes in tissue structure, moisture loss, lipid oxidation and protein degradation during frozen storage.

Nile Tilapia *(Oreochromis Niloticus)* is an extensively farmed fish. Because of its excellent biological characteristics and edible value, it becomes a popular food material around the world^1^. However, like other aquatic products, tilapia is highly perishable^2^. Generally, the most common and effective method to maintain its freshness is frozen storage^3^. Low temperatures can delay the deterioration of fish, but it also inevitably brings negative impacts on fish tissue^4^. During frozen storage, composition changes and moisture loss result in a honeycomb microstructure in the fish tissue. Such porous structure is inherently irregular and difficult to describe^5^.

The fractal dimension is a geometry parameter characterized as the space-filling capacity of a space structure at a certain scale^5^, which can be used to describe the porous structure in some food materials including frozen tilapia^6^. Fractal dimension analysis was also used as a quantitative indicator reflecting the surface roughness of frozen tofu^7^. In addition, Kerdpiboona *et al*. established a fractal dimension model for describing the relationship between microstructural and physical properties of dried foods^8^.

In this study, the preservation effects of tangerine peel (TP, *Citri reticulatae pericarpium*)^9^ on the tissue structure and qualities of frozen tilapia were evaluated using fractal dimension along with measurements of changes in hardness and springiness, as well as chemical compositions due to moisture loss, lipid oxidation and protein decomposition. The results demonstrated the applicability and accuracy of fractal dimension analysis for predicting qualities and shelf life of frozen tilapia.

## Materials and Methods

All methods in this study were carried out in accordance with relevant guidelines and regulations. All experimental protocols were approved by South China University of Technology.

### Preparation of TP extract

Fresh TP (obtained from Huping Mountain, Hunan Province, China) was dried in a convection oven at 50 °C for 48 h and hydro-distilled in a Clevenger-type apparatus (Kesijia Ltd., Beijing, China) for 6 h to obtain the extract^10^. The resulting extract was dried with hydrous sodium sulfate, filtered, and stored at 4 °C in brown sealed glass vials.

### Sample preparation and preservation

Tilapia, with average weight of 447 ± 63 g, was obtained from Lianfeng Aquaculture Base of Guangzhou, Guangdong Province, China. The fish was kept in plastic tanks at 25 °C for 24 h. Then it was killed, skinned, processed into butterfly-like fillets and thoroughly washed in dilute NaCl solutions. The resulting fillets were divided into 6 groups. Three of them were control groups without TP treatment; the other three groups were treated with TP extract by immersing the samples in an aqueous solution containing 0.2% *v/v* TP extract at ~15 °C for 30 min. These samples were frozen and stored in freezers (BCD-235NCQE, Le-jin Company, China) at −4 ± 0.2 °C, −8 ± 0.4 °C, and −18 ± 0.5 °C, respectively, for 40 days. Samples were taken every 10 days from the freezers for structural and chemical analyses. Each measurement was performed with at least three repeats in three different samples. The results were obtained by averaging. The data was processed using SPSS 17.0 for statistical analysis. One way analysis of variance was conducted to compare the effects under different storage times (*p* < 0.05). The least significance difference (LSD) test was used to determine differences at *a* = 0.05^11^.

### Microscopic tissue imaging and fractal dimension analysis

The micro-sections of tilapia samples were prepared using the method described by He *et al*.^6^. The fillets were imbedded in frozen OCT (optimal cutting temperature) agents and cut into 20 μm slices using a freezing microtome (Model CM 1900; Leica Co. Ltd, USA). Then, the slices were stained with H&E method and images were obtained under an optical microscope (BX51; Olympus, USA). Fractal dimension values were obtained by the box-counting method^12^. The optical microscope image of a tissue sample was divided into square sub-boxes with a variable length denoted as *ε*. The total number of sub-boxes with more than 50% of its tissues was then counted as *N*(*ε*). The relationship between *ε* and *N* (ε) can be described by the following equation:


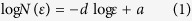


where *d* is the fractal dimension of the divided image and *a* is a constant. By plotting log *N(ε)* vs. log *ε,* the fractal dimension *d* can be obtained from the slope of the regression line (see Fig. S1 in Supplemental Materials).

### Texture profile analysis

Texture profile analysis (TPA) was performed under the conditions described in a similar study^13^. A 6 mm cylindrical probe was used with 5 g trigger load and 5 mm/s test speed. Twice compressions with 5 mm depth were performed on the same point of each sample and separated by a 5 s interval. The TPA parameters including hardness and springiness were obtained from the measured force (N), area (Ns) and distance (mm) between peak heights.

### Moisture content

The moisture content (MC) was estimated from the weights before and after drying to a constant weight in a 105 °C oven^14^. Water activity (A_w_) was measured with minced tilapia tissue in a portable A_w_ instrument (Hygro Palm, Rotronic Company, Switzerland)^15^.

### Lipid oxidation

Peroxide value (PV) was measured using the method reported by Lea^16^. Fifty grams of tilapia were minced and extracted with a mixture of 50 mL distilled water, 100 mL methanol, and 100 mL chloroform. After dissolving 1 g liquid extract in a mixture of 10 mL chloroform and 15 mL acetic acid, 1 mL saturated potassium iodide was added and the mixture was kept in the dark for 10 min. Then, 30 mL distilled water and 1 mL starch solution (1% w/v) were added, and the solution was titrated with 0.01 mol equiv/L Na_2_S_2_O_3_ until colorless. The PV was calculated from the dosage of Na_2_S_2_O_3._

Thiobarbituric acid (TBA) was analyzed as described by Li *et al*. with some modifications^17^. One gram of tilapia was minced, dissolved in 5 mL of 4% v/v 1-butanol aqueous solution, pipetted into a dry stoppered test tube, mixed with 5 ml TBA reagent (200 mg 2-TBA dissolved in 100 mL 1-butanol), and incubated in a water bath at 95 °C for 120 min. After cooling, the absorbance at 530 nm of the mixture was measured to calculate the TBA.

### Protein decomposition

Total volatile basic nitrogen (TVB-N) was measured using the semi-micro Kjeldahl method^18^. Ten grams of tilapia were minced, mixed with 50 mL distilled water, stirred for 30 min and then filtered. The filtrate was alkalinized with 10% suspension liquid of MgO and submitted to a Kjeldahl Apparatus (KDY-9820, Beijing, China). The volatile basic nitrogen or ammonia was absorbed in an acid receiver and determined by titration with 0.01 M HCl solution.

Trichloroacetic acid (TCA) soluble peptides were analyzed using the Lowry method^19^. Three grams of tilapia were minced and homogenized with 27 mL cold 5% (w/v) TCA. The homogenate was kept in ice for 30 min and centrifuged at 10000 rpm for 5 min at 4 °C. The soluble peptides in the supernatant were then determined and expressed as mmol tyrosine/g muscle.

## Results and Discussion

### Effects of frozen storage on tilapia tissue structure and texture

**Figure 1:**
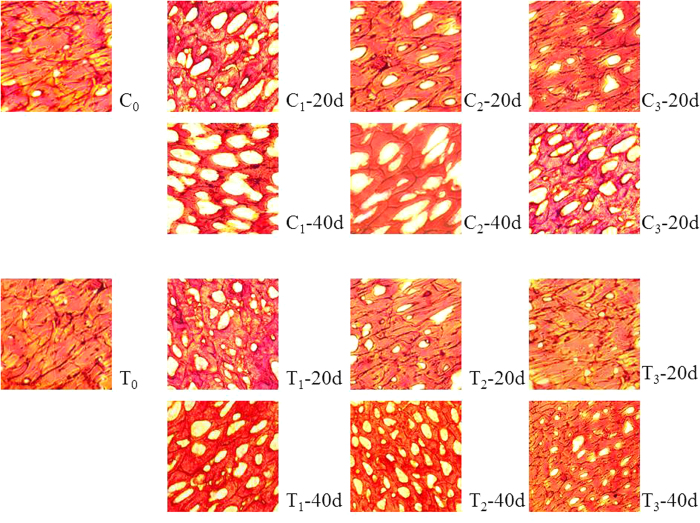
The tissue structure of tilapia sample during storage. C_1_, C_2_ and C_3_: the control sample stored at −4 °C, −8 °C and −18 °C. T_1_, T_2_ and T_3_: the extracts treating sample stored at −4 °C, −8 °C and −18 °C. 20d and 40d: the storage time of 20 day and 40 day.

Changes in tilapia tissue structure and morphology during frozen storage at −18 °C (a common temperature for industrial storage), −8 °C (a temperature often used in consumers’ fridge), and −4 °C (a usual temperature for partially frozen storage)^20^ were studied. These three temperatures are representative in aquatic products storage^21^. Figure 1 shows the microscopic images of micro-sections of tilapia tissues after 20 days and 40 days at −4 °C, −8 °C and −18 °C. Compared to the initial samples, there were increasing void spaces (white) inside the frozon tissue (red) during storage^22^. Initially, tilapia tissues had only a few small void spaces, which grew into larger irregular pores over time^23^, which could be attributed to fluids migration caused by the ice crystallization and muscle fiber disarrangement caused by protein decomposition^6^. The increases in the void spaces were faster at higher temperatures. Interestingly, TP extract coating significantly hindered the structural change and was able to maintain tilapia tissue with fewer and smaller void spaces, especially at −18 °C.

**Figure 2:**
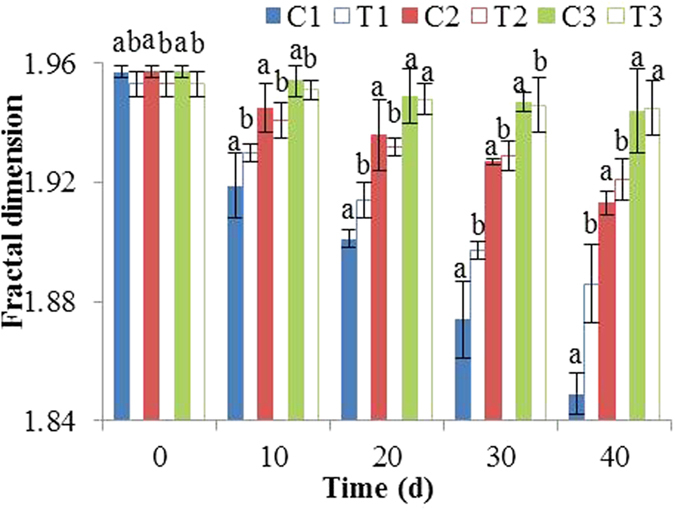
The variations in fractal dimension of sample structure during 40-day storage. C_1_, C_2_ and C_3_: the control sample stored at −4 °C, −8 °C and −18 °C; T_1_, T_2_ and T_3_: the extracts treating sample stored at −4 °C, −8 °C and −18 °C.

Changes in tilapia tissue structure can be quantitatively analyzed by measuring the fractal dimension, which is sensitive enough to distinguish tiny differences in the structure, size, or area fraction^24^,^25^. Figure 2 shows the fractal dimension values of tilapia samples at various frozen temperatures and storage times. In general, fractal dimension decreased with increasing the temperature and storage time, and the decrease was significantly slower for samples treated with TP extract.

**Figure 3:**
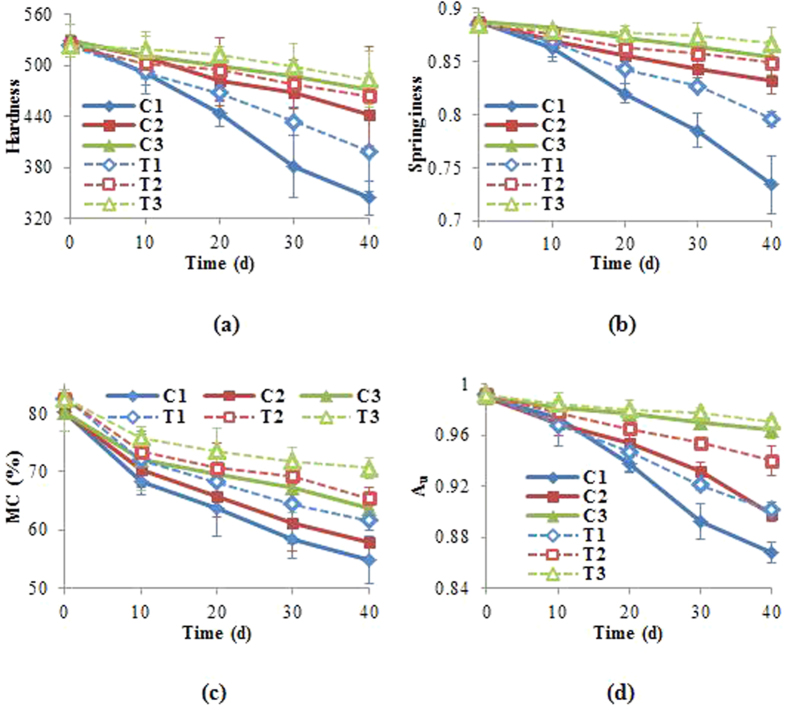
The variations in texture and moisture characteristics of sample during 40-day storage. (**a**) Hardness; (**b**) Springiness; (**c**) MC and (**d**) A_w_; C_1_, C_2_ and C_3_: the control sample stored at −4 °C, −8 °C and −18 °C; T_1_, T_2_ and T_3_: the extracts treating sample stored at −4 °C, −8 °C and −18 °C.

Changes in the tissue structure of tilapia should also affect its texture properties^7^. As shown in Fig. 3a,b, both hardness and springiness of frozen tilapia tissues also decreased over time, in similar trends to those for fractal dimension. Clearly, the increasingly porous structure, as quantified by fractal dimension, formed during frozen storage resulted in the declines in hardness and springiness. As expected, TP extract treatment slowed down the declines in these texture properties.

### Changes in moisture content during frozen storage

**Figure 4:**
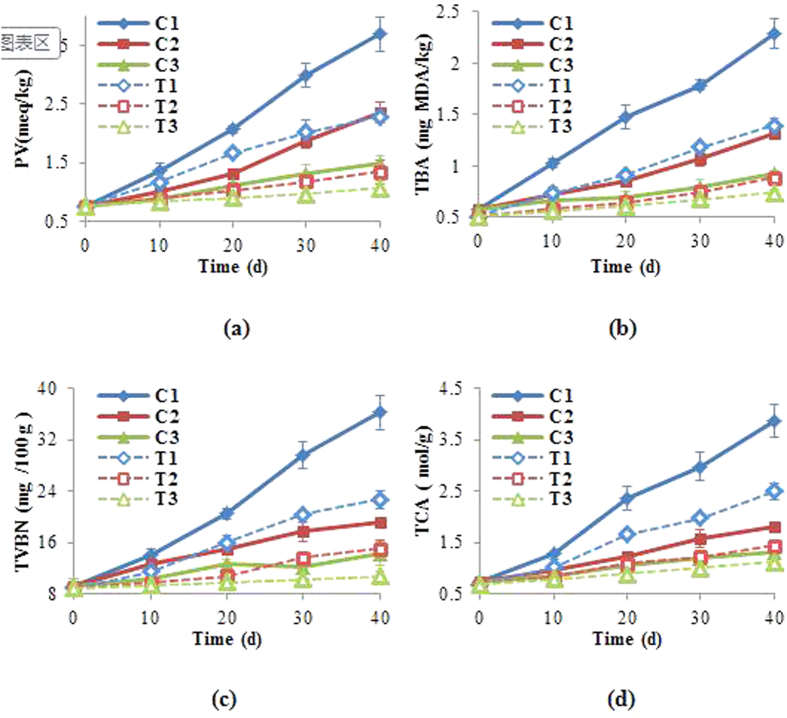
The variations in chemical composition of sample during 40-day storage. (**a**) PV; (**b**) TBA; (**c**) TVB-N and (**d**) TCA. C_1_, C_2_ and C_3_: the control sample stored at −4 °C, −8 °C and −18 °C. T_1_, T_2_ and T_3_: the extracts treating sample stored at −4 °C, −8 °C and −18 °C.

Variations in tissue structure and texture of tilapia tissue are greatly affected by the moisture content and chemical compositions^26^. During frozen storage, the crystallized tissue fluids greatly destroyed the structure of the sample tissue^27^, while the loss of moisture due to sublimation created the honeycomb microstructure with large void spaces in the muscle tissue^28^. Figure 3c,d shows changes in the moisture content (MC) and water activity in frozen tilapia tissues. After 40 days, MC in the control group reduced 25.3%, 22.3% and 16.4% at −4 °C, −8 °C and −18 °C, respectively, from the initial value of 80.18 ± 3.15%. In contrast, tilapia tissues treated with the TP extract lost only 19.0%, 17.2% and 12.0%, respectively. Similar decreasing trends in A_w_ were also observed (Fig. 4b). The classical Brunauer–Emmet–Teller model has been used to describe the close relationship between fractal dimension and moisture content^29^,^30^. Based on the fractal generalization of the Frenkel-Holsi-Hill (FHH) equation, Pfeifer *et al*. showed that the dimension of fractal surface *d*_*fs*_ was governed by the slope of the linear plot of moisture content vs. *ln h*, where *h* was the relative pressure^31^. It is thus clear that moisture lost during frozen storage can be quantitatively monitored through changes in fractal dimension.

### Changes in chemical composition

Protein and lipid are two major organic components in fish tissue^32^. A chemical composition analysis showed that fresh tilapia tissue contained 14.70 ± 0.63% protein and 2.94 ± 1.19% lipid. Changes in protein and lipid contents in frozen tilapia also occurred during storage, which would reduce the water-retaining capability of the tissue and result in faster moisture loss. It can also directly disarrange muscle fibers and change tissue structure and texture^33^,^34^.

### Lipid oxidation

Changes in the lipid content usually can be quantified by monitoring the degree of lipid oxidation, which is a significant process of chemical deterioration in the refrigerated aquatic products^35^. In this process, free radicals “stolen” electrons from the polyunsaturated fatty acids which contain multiple double bonds in cell membranes. It impacts water-retaining capability of tissue and results in the variations in the tissue structure^36^,^37^. PV and TBA are two common parameters for determining the degree of lipid oxidation^38^. In general, both PV and TBA in frozen tilapia increased with the storage time (Fig. 4a,b). The tilapia tissue had the initial PV of ~0.76 meq/kg, which increased to 3.69, 2.35, and 1.48 meq/kg in the control samples after 40 days at −4 °C, −8 °C and −18 °C, respectively (Fig. 4a). In contrast, the TP extract treated tilapia tissues had significantly lower PV of 2.29, 1.34 and 1.06 meq/kg at the respective storage temperatures. Similar trends were also observed with TBA, which increased from the initial 0.58 mg MDA/kg to 2.29, 1.31, and 0.93 mg MDA/kg for the control and from 0.51 MDA/kg to 1.39, 0.88, and 0.74 mg MDA/kg for the treated samples after 40 days at −4 °C, −8 °C and −18 °C, respectively (Fig. 4b). Since lipid oxidation is mainly associated with unsaturated fats exposed to oxygen or air, TP extract as a protective barrier on the sample surface can slow down lipid oxidation by deterring the contact with oxygen.

### Protein decomposition

TVB-N and TCA soluble peptides are parameters useful for monitoring protein decomposition^31^. Both TVB-N and TCA soluble peptides in frozen tilapia increased significantly during frozen storage (Fig. 4c,d). As expected, the increases were slower at lower temperatures and with TP extract treatment. The muscle fiber in tilapia tissue composed mainly proteins, and therefore protein decomposition would greatly affect tissue morphology and texture^39^. Accordingly, changes in TVB-N and TCA soluble peptides would also affect the fractal dimension.

### Fractal dimension as the key parameter for shelf-life prediction

Pearson correlation coefficient, which is widely used to determine the degree of linear dependence between two variables^6^, was calculated to determine correlations between fractal dimension and various texture and chemical parameters during frozen storage of tilapia (see Fig. S2 in Supplemental Materials). Table 1 shows that all have a Pearson correlation coefficient of ±0.95. This result indicated that fractal dimension can be employed as a reliable quality index to reveal variations in chemical and texture parameters quantitatively.

The shelf-life of the tilapia samples was predicted by first-order kinetics model with using fractal dimension of tilapia tissues as the key quality factor^40^. The model can be expressed as Eq. 2.


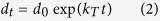


where *t* is the storage time (d), *d*_*0*_ is the initial value of fractal dimension, *d*_*t*_ is the fractal dimension value at storage time *t*, and *k*_*T*_ is the rate constant at storage temperature *T*.

Additionally, the relationship between the rate constant *k*_*T*_ and the storage temperature *T* can be shown by the Arrhenius equation as follows^41^:


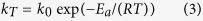


where *k*_0_ is the pre-exponential factor (d^−1^), *E*_a_ is the activation energy (kJ/mol), and *R* is the gas constant (8.314 J/mol K). *T, k*_*T*_*, k*_*0*_, and *E*_*a*_ are constants associated with the physical nature of the reaction system.

Incorporating Eq. 2 with Eq. 3, a global equation can be formulated:


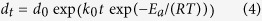


With using the data of fractal dimension in the Fig. 2, activation energy *E*_*a*_ of the control samples was calculated as −1.104 × 10^13^ kJ/mol, and pre-exponential factor *k*_*0*_was 9883.975 d^−1^. Meanwhile, *E*_*a*_ of treated samples was −2.343 × 10^13^ kJ/mol, and *k*_*0*_was 10179.648 d^−1^. Accordingly, the first-order kinetics equation of fractal dimension was obtained as follows:


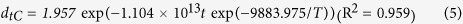



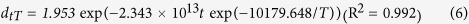


In addition, the maximum of fractal dimension in spoiled tilapia samples were 1.801 ± 0.025^6^. So the results of the shelf-life were obtained as listed in Table 2. The data was harmonious with similar researches^6^,^40^,^41^, which indicated that fractal dimension employed as a quality index was reliable. Additionally, the TP extracts treatment prolonged the shelf-life of the frozen tilapia about 40%. The results can confirm and quantify the effects of TP extracts on freshness of preserved fish reported in a previous study^9^.

### Effects of TP extract

The result of GC-MS analysis of TP extracts was listed in Table S1. Limonene (68.44% w/w) and γ-terpinene (18.39% w/w) made up 85% of total TP extract. Limonene has been proved as an effective agent to inhibit the activity of spoilage bacteria, and its molecule containing multiple double bonds, which can be an effective barrier against lipid oxidation as an alternative of polyunsaturated fatty acids to offer electronics^42^. γ-terpinene was also recorded as an important preservative agent because of its antibacterial and antioxidant effects to some extent^2^. Additionally, many other compounds was known to be able to play positive roles to maintain the chemical properties of preserved materials^9^,^43^,^44^,^45^,^46^. Therefore, as shown in Fig. 4, TP extract showed good preservation effect for frozen-stored tilapia with significant inhibition effects on of chemical degradation. The effects can accordingly reduce the variation in tissue structure and resulting fractal dimension of tilapia.

## Conclusion

In this study, the frozen fish tissues with TP extracts treatment were analyzed for structural and chemical changes. During frozen storage, fractal dimension, which quantifies the porous structure formed in the tissue samples, as well as texture properties including hardness and springiness, and moisture content and water activity all decreased during the storage, while the extents of lipid oxidation, measured as peroxide value and thiobarbituric acid concentration, and protein degradation, monitored with total volatile basic nitrogen and trichloroacetic acid soluble peptides increased. The change rates of these parameters decreased with decreasing the storage temperature and by applying TP extract. With excellent correlations among each other, fractal dimension can reveal the variations of chemical parameters as well as texture parameters accurately. A shelf life prediction with using fractal dimension as the quality index demonstrated that TP extract could extend the shelf life of frozen tilapia by 35–45%.

## Additional Information

**How to cite this article**: He, Q. *et al*. Quality Evaluation Focusing on Tissue Fractal Dimension and Chemical Changes for Frozen Tilapia with Treatment by Tangerine Peel Extracts. *Sci. Rep.* **7**, 42202; doi: 10.1038/srep42202 (2017).

**Publisher's note:** Springer Nature remains neutral with regard to jurisdictional claims in published maps and institutional affiliations.

## Supplementary Material

Supplementary Table and Figures

## Figures and Tables

**Table 1:** Pearson correlation coefficients of fractal dimension in relation to texture and chemical indexes.

	Texture				Chemical indexes			
	Hardness	springiness	MC	A_w_	PV	TBA	TVB-N	TCA
C1	0.987	0.979	0.985	0.975	−0.988	−0.994	−0.983	−0.982
C2	0.986	0.999	0.994	0.986	−0.965	−0.970	−1.000	−0.988
C3	0.998	0.991	0.966	0.996	−0.990	−0.987	−0.956	−0.979
T1	0.971	0.970	0.987	0.978	−0.986	−0.972	−0.965	−0.978
T2	0.973	1.000	0.946	0.993	−0.987	−0.966	−0.944	−0.996
T3	0.920	0.952	0.950	0.970	−0.976	−0.980	−0.987	−0.982
AVE	0.973	0.983	0.971	0.983	−0.982	−0.978	−0.973	−0.984

(C_1_, C_2_ and C_3_: the control sample stored at −4 °C, −8 °C and −18 °C. T_1_, T_2_ and T_3_: the extracts treating sample stored at −4 °C, −8 °C and −18 °C).

**Table 2:** Predicted shelf-life of frozen tilapia stored at different temperatures.

T/°C	Control/d	Treated/d	Increase/%
−2	52.02	70.41	35.34%
−4	68.23	93.09	36.43%
−6	89.85	123.58	37.55%
−8	118.80	164.77	38.69%
−12	210.42	296.82	41.06%
−18	512.93	742.89	44.83%
