# Supplementary material for: Quality Evaluation Focusing on Tissue Fractal Dimension and Chemical Changes for Frozen Tilapia with Treatment by Tangerine Peel Extract
Source: Sci Rep. 2017 Feb 7;7:42202. doi: 10.1038/srep42202 (PMC5294564; doi:10.1038/srep42202)
Supplement: Supplementary Table and Figures [file srep42202-s1.pdf]

Quality Evaluation Focusing on Tissue Fractal Dimension and Chemical Changes for Frozen Tilapia with Treatment by Tangerine Peel Extract

Author

Qi He[1]; Zhao Yang[2]; Bin Gong[1]; Jingjing Wang[1]; Kaijun Xiao[1*]; Shang-Tian Yang[3*]

Author affiliation

1. College of Food Science and Engineering, South China University of Technology, Guangzhou city, Guangdong province, China, 510640.

2. Guangdong Food and Drug Vocational College, Guangzhou city, Guangdong province, China, 510520.

3. Department of Chemical and Biomolecular Engineering, The Ohio State University, Columbus, Ohio, US, 43210.

Corresponding authors

[*]Kaijun Xiao, E-mail: fekjxiao@scut.edu.cn; telephone: 86-20-87113843.

[*]Shang-Tian Yang, E-mail: yang.15@osu.edu; telephone: 1-614-292-6611.

Table S1.

Analysis of main components (more than 0.15% w/w) present in the TP extract.

RI	Components	Weight % (w/w)
832	Furfural	0.15
937	α-Pinene	1.34
952	Camphene	0.29
974	Sabinene	0.22
981	β-Pinene	0.51
991	β-Myrcene	2.06
1010	α-Phellandrene	0.2
1022	2-Carene	0.17
1030	Limonene	68.44
1059	γ-Terpinene	18.39
1185	α-Terpinolene	0.52
1094	Linalool	3.66
1005	Octanal	0.5
1203	Decanal	0.28
1506	α-Farnesene	0.25
	Total	96.98

All compounds were identified and confirmed using the Kovats indices as described by Rambla et al.[11] Weight % was determined from the peak area relative to the total peak area. RI is the retention index.

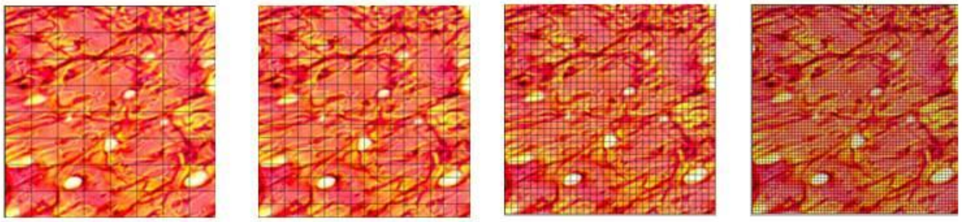

ε	1/8	1/16	1/32	1/64
$-\log \varepsilon$	0.903	1.204	1.505	1.806
N	64	256	1028	4096
$N(\varepsilon)$	64	249	967	3743
$\log N(\varepsilon)$	1.8062	2.3962	2.9854	3.5732

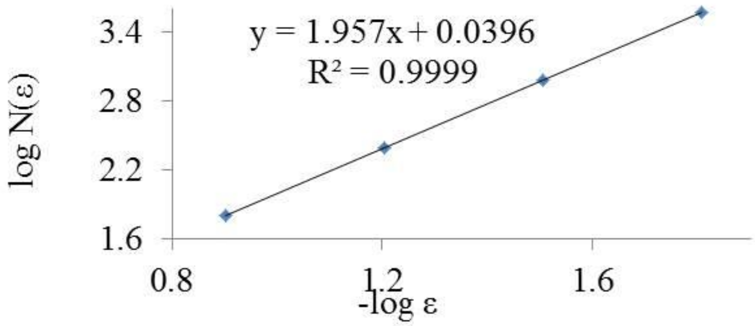

Fig. S1. Determination of fractal dimension using the box-counting method. (The values of ε were set up to 1/8, 1/16, 1/32, and 1/64 of the image length).

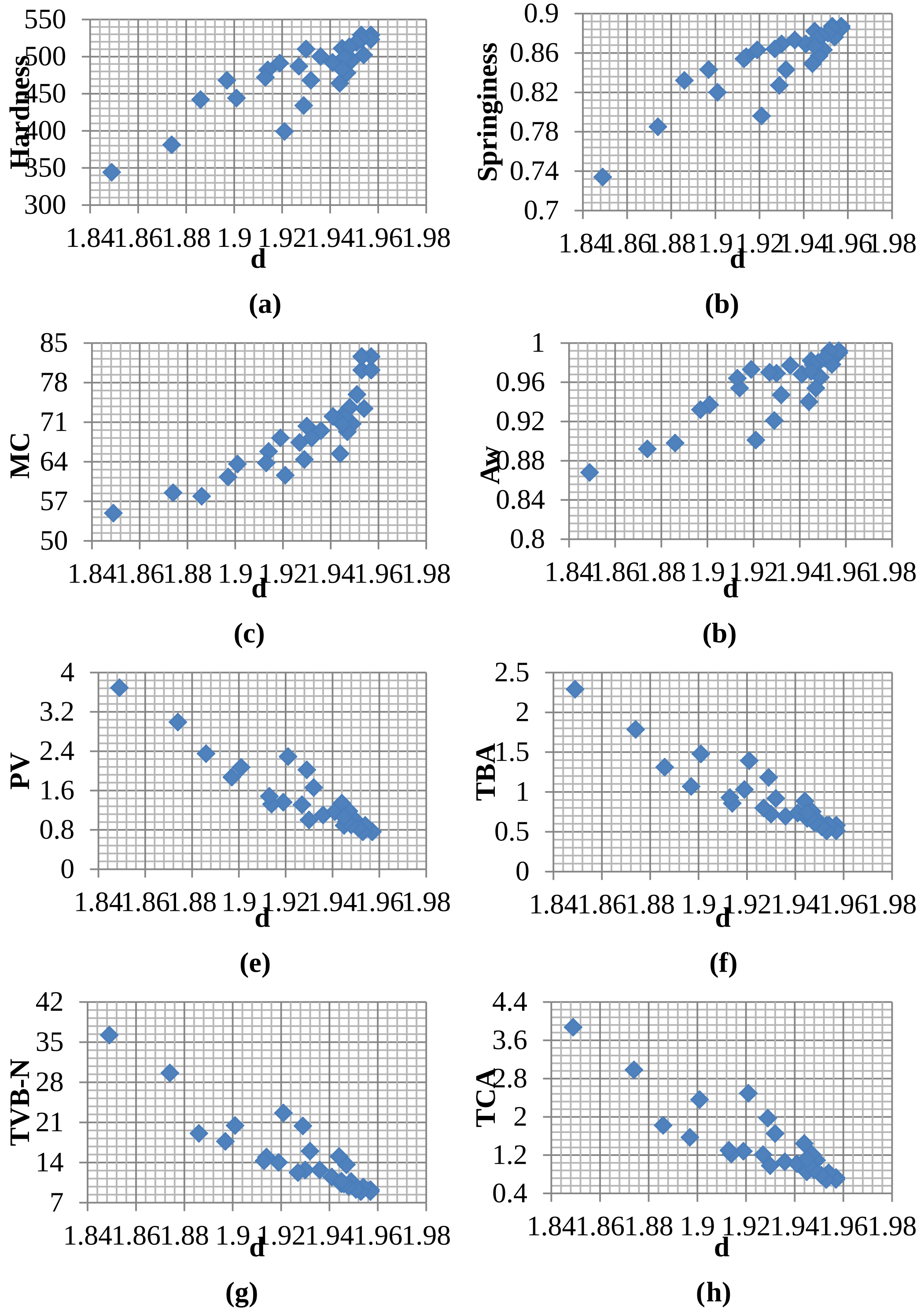

Fig. S2. Correlation of fractal dimension in relation to texture and chemical indexes. (a)Hardness; (b)Springiness; (c)MC; (d)Aw; (e)PV; (f)TBA; (g) TVB-N and (h) TCA.
